# Free Extracellular miRNA Functionally Targets Cells by Transfecting Exosomes from Their Companion Cells

**DOI:** 10.1371/journal.pone.0122991

**Published:** 2015-04-29

**Authors:** Krzysztof Bryniarski, Wlodzimierz Ptak, Emilia Martin, Katarzyna Nazimek, Marian Szczepanik, Marek Sanak, Philip W. Askenase

**Affiliations:** 1 Section of Allergy and Clinical Immunology, Department of Internal Medicine, Yale University School of Medicine, New Haven, Connecticut, United States of America; 2 Department of Immunology, Jagiellonian University Medical College, Krakow, Poland; 3 Department of Medical Biology, Jagiellonian University Medical College, Krakow, Poland; 4 Department of Internal Medicine, Jagiellonian University Medical College, Krakow, Poland; Mie University Graduate School of Medicine, JAPAN

## Abstract

Lymph node and spleen cells of mice doubly immunized by epicutaneous and intravenous hapten application produce a suppressive component that inhibits the action of the effector T cells that mediate contact sensitivity reactions. We recently re-investigated this phenomenon in an immunological system. CD8+ T lymphocyte-derived exosomes transferred suppressive miR-150 to the effector T cells antigen-specifically due to exosome surface coat of antibody light chains made by B1a lymphocytes. Extracellular RNA (exRNA) is protected from plasma RNases by carriage in exosomes or by chaperones. Exosome transfer of functional RNA to target cells is well described, whereas the mechanism of transfer of exRNA free of exosomes remains unclear. In the current study we describe extracellular miR-150, extracted from exosomes, yet still able to mediate antigen-specific suppression. We have determined that this was due to miR-150 association with antibody-coated exosomes produced by B1a cell companions of the effector T cells, which resulted in antigen-specific suppression of their function. Thus functional cell targeting by free exRNA can proceed by transfecting companion cell exosomes that then transfer RNA cargo to the acceptor cells. This contrasts with the classical view on release of RNA-containing exosomes from the multivesicular bodies for subsequent intercellular targeting. This new alternate pathway for transfer of exRNA between cells has distinct biological and immunological significance, and since most human blood exRNA is not in exosomes may be relevant to evaluation and treatment of diseases.

## Introduction

Hapten applied epicutaneously (ec) induces effector T cells that mediate late phase of contact sensitivity reaction (CS) and triggers B1a lymphocytes to produce specific IgM antibodies and their light chains (Ab LC), involved in CS early phase as shown by us previously [1]. In contrast, intravenous (iv) hapten injection generates suppressor CD8^+^ T cells (Ts) that inhibit the action of effector T cells mediating CS reactions. Interestingly, lymphoid cells of mice tolerized by double immunization (i.e. iv and then ec) produce a suppressor factor (TsF) in vivo and in vitro that acts similarly to suppressor T cells from antigen tolerized mice (Ts) and was formerly described as consisting of two essential components originating from respective immunizations [2]. One of the components is produced by CD8^+^ Ts cells generated by iv hapten application and the other by B1a lymphocytes induced by skin painting [1,3]. As found recently, the induced CD8+ Ts cells release miR-150 containing exosomes that also require the presence of exosome-bound B1a cell products (i.e. antibody light chains, Ab LC) to act as TsF. As shown in many systems, exosomes secreted by various cells contain their membranes and cytoplasmic contents (proteins, RNA), which they can transfer to target cells and thus play a significant role in intercellular communication by affecting functional changes in the acceptor cells [4]. Therefore, these suppressive exosomes that down-regulate CS reaction are a combined product of T cells (the exosomes containing miR-150) and B cells (surface Ab LC).

We used the model system of T cell-mediated immunity in mice known as cutaneous CS. In this model the mechanism of immunological tolerance, mediated by Ts cells inhibiting effector T cells, was explained in our prior studies by nanovesicle transfer of functional extracellular RNA (exRNA) between cells [5–7]. We concluded that these nanovesicles meet a variety of criteria to be referred to as exosomes [8–10] whose features were characterized previously [5,11]. Studied exosomes are present in plasma of tolerized mice and in the culture supernatant of their lymphoid cells containing CD8+ Ts cells. Uniquely in this system, the suppressive exosomes act antigen (Ag)-specifically due to a surface coat of Ab LC [5] produced by B1a cells activated during the tolerogenesis and accompanying suppressor CD8+ T cells. Thus, B1a cells are present both, in the tolerized and effector cell mixtures, since they are also required to elicit positive CS responses [6,7,12,13].

Exosome-producing CD8+ Ts lymphocytes are not classical FoxP3^+^ Treg cells and act in vitro and in vivo to inhibit both CD4+ or CD8+ CS-effector T cells by transferring miR-150 [5], also associated with many other T cell functions [14–18]. The suppression is likely caused by the Ab LC coated exosomes targeting the antigen presenting cells supporting the effector T cells [19]. Such transfer of functional genetic information by passage of miRNA between cells in vesicles is a new paradigm with far reaching consequences for homeostasis maintenance and in the pathogenesis of many diseases, such as cancers, as well as allergies, autoimmunity and other immunological and inflammatory diseases [20,21].

Although exRNA can be transferred by exosomes, the significant amount of exRNA is also present in circulating body fluids. Our current study investigated if such freely circulating exRNA (hereafter called free exRNA) could also be functionally active in the tolerance mechanism. We showed that free exRNA without exosome carrier also mediated Ag-specific suppression due to the delivery of miR-150. This puzzling finding was demonstrated to be a result of exRNA association with exosomes produced by B1a cells accompanying the finally targeted effector T cells in the CS-mediating cell mixture. To our knowledge, this is the first demonstration of functional transfer of free miRNA and additionally its inhibition of target cells by associating with exosomes from their companion cells. This represents an alternate pathway of free exRNA intercellular transfer that contrasts with donor cell release of RNA-containing, cell-targeting exosomes. Since significant amounts of circulating exRNA are not in exosomes, these findings likely have important biological and immunological significance, and may be relevant to both, pathogenesis and treatment of diseases.

## Materials and Methods

### Animals and Ethics Statement

CBA/J, C57BL/6, and TLR-3^-/-^ mice were obtained from the NCI or Jackson Labs. CBA/J mice were also from the breeding unit of the Department of Immunology, Jagiellonian University, Medical College in Krakow, Poland. Mice were fed autoclaved food and water.

#### Animal Research

This study was carried out in strict accordance with the recommendations in the Guide for the Care and Use of Laboratory Animals of the National Institutes of Health. The protocols were approved by the Institutional Animal Care and Use Committee of the Yale University, New Haven, CT, (Permit Number: 07381) and 1-st Local Ethics Committee of the Jagiellonian University, Krakow, Poland (Permit numbers: 64/2008, 40/2011 and 106/2012). All procedures were performed under ether or isoflurane anesthesia, and all efforts were made to minimize suffering.

### Reagents

Picryl chloride (PCl/ trinitrophenyl (TNP)-Cl), oxazolone (OX), EDTA sodium salt, 2-mercaptoethanol (2ME), 3-(4,5-dimethylthiazol-2-yl)-2,5-diphenyltetrazolium bromide (MTT), trinitrobenzene sulfonic acid (TNBSA), cacodylic acid, sodium acetate, trypan blue, phenol-chloroform pH 4.7, phenol equilibrated with Tris-HCl buffer pH = 8.0, RNase A (4375 and purer 5250), DNase (D7231, Lot K1815), Proteinase K (P2308, Lot 65u8603), human recombinant IL-2, all were from [Sigma, St. Louis, MO]. ShortCut RNase III [New England Biolabs, Ispwich, MA]; chloroform, acetone [Baler, Philipsburg, NJ]; glycogen for molecular biology, sodium dodecyl sulfate (SDS) [Roche Diagnostic GmbH, Manheim, Germany]; low toxic rabbit complement [Pel-Freeze Biologicals, Brown Deer, WI]; anti-CD8a microbeads (cat. No 130-049-401) [Miltenyi Biotec, San Diego, CA]; Trypsin (0152–17, Lot 576331) [DIFCO Laboratories, Detroit, MI]; 8-hydroxyquinoline [Fisher Scientific Corp., Fairlawn, NJ]; ethanol [Pharmaco-Aaper, Brookfield, CT]; MOPS [USB, Cleveland OK]; RNase free water, ethidium bromide [American Bioanalytical, Natick, MA]; Qiagen DNA/RNA Maxi kit (cat No. 14162) [Qiagen. Valencia, CA]; agarose low mw Cat No. 162–0102 [BioRad, Hercules, CA] anti-miRs to miR-150 and miR-150* and other miRNA [Thermo Scientific Dharmacon; Fisher RNAi technologies, Lafayette, CO] were obtained from the suppliers.

### Culture media

Mishell-Dutton Medium (MDM), RPMl1640 and Minimal Essential Medium Amino acids (MEM), [Sigma, St. Louis, MO]; Hepes solution [American Bioanalytical, Natick, MA]; Dulbecco’s Phosphate Buffered Saline (DPBS), Dulbecco’s Modified Eagle Medium (DMEM), Pen-Strep, Sodium pyruvate, L-Glutamine all from [Gibco Invitrogen, Auckland NZ]; Sequagel [National Diagnostic, Manville, NJ], were obtained from the manufacturers.

### Antibodies

Rat anti-mouse anti-CD3 IgM mAb (line C363-28b), rat anti-mouse anti-CD4 IgG2b mAb (line GK1.5), rat anti-mouse anti-CD8 IgG2b mAb (line TIB-105) were obtained as generous gifts from the Immunobiology Laboratory of Charles Janeway Jr at Yale and used at concentrations established in Janeway’s Laboratory.

### Contact Sensitization. Active Immunization and Adoptive Transfer of CS-effector cells treated with exRNA

Under light ether or isoflurane anesthesia, mice were optimally contact sensitized by application to the shaved abdomen, chest and feet skin of 150μl of 5% picryl chloride (PCl, TNP-Cl), or 3% oxazolone (OX) in ethanol: acetone (3:1) [5,6,22]. CBA/J mice are susceptible to both PCl and OX sensitization, in contrast to C57BL/6 mice that develop effective CS only after contact immunization with OX. For adoptive transfer, on day 4 actively contact sensitized mice were sacrificed by cervical dislocation and spleens and axillary and inguinal lymph nodes were harvested to obtain sensitized cells. Single cell suspensions (7 x 10^7^ per recipient) of these CS-effector cells treated with appropriately processed fractions of Ts Sup (phenol chloroform extract—(PCE), Qiagen column derived RNA fraction (QRNA), Qiagen column derived DNA fraction (QDNA), enzyme-digested samples or related negative control fractions, used in a dose dependent on experiment protocol, standardly 3ml of Ts Sup, 15μg of PCE, 3μg of QRNA or 3μg of QDNA per recipient) as well as untreated immune cells as a positive control, were used for iv adoptive cell transfer to naïve recipients, that were lightly anesthetized and then immediately challenged on both sides of both ears with 0.4% TNP-Cl or OX in acetone: olive oil (1:1). In case of active CS, contact sensitized animals were only challenged in analogous manner. Subsequent increase in ear thickness was measured basically 24 h later (CS late phase manifestation) and in active sensitization also 2 h later for evaluation of early CS phase with an engineer’s micrometer (Mitutoyo) and expressed in units of 10^-2^ mm ± SE [5,22]. Background non-specific increase in ear thickness (about 1–2 units at 24h) of similarly TNP-Cl or OX ear challenged non-sensitized littermate control animals, was subtracted from results of each experimental group to yield the net ear swelling response, expressed in units x 10^-2^ mm.

### Induction of Hapten-Specific T Suppressor Cells (TNP Ts or OX Ts), Ts Supernatant (TNP Ts Sup, OX Ts Sup), and Control Normal Cell Supernatant (Nl Cell Sup)

For tolerance induction, freshly prepared syngeneic mouse red blood cells (mRBC) were conjugated with hapten (TNP or OX) [5], and then injected in 0.25ml of 10% solution iv at day 0 and 4 into naïve mice that were then skin contact painted (immunized) with homologous hapten at day 9. Spleens and lymph nodes were harvested at day 11 from tolerized mice and used as a source of Ts cells that were cultured in serum free Mishell-Dutton medium supplemented with pyruvic acid, glutamine and MEM additional amino acids, 2-ME, hepes and antibiotics (pen-strep) at a concentration 2 x 10^7^ per ml for 48 h to obtain suppressive Ts Sup [22], shown previously to contain hapten-specific suppressive exosomes [5]. For control Nl Cell Supernatant, cells were harvested from spleens and lymph nodes of naive mice and cultured as above. Further assessment of activity of various fractions obtained from Ts cell Sup is summarized in S9 Fig.

### Negative and Positive Selection of Ts Cell Populations

Ts cells were treated with monoclonal antibodies (mAb) specific for either CD3, CD4 or CD8 markers in the presence of rabbit complement for 1 h in 37°C water bath, followed by assessment of cell viability with trypan blue. Then negatively selected cells were cultured in vitro for 48 h to obtain Ts Sup from various remaining cell fractions in standard conditions described above.

For positive selection, magnetic beads (Miltenyi) coated with anti-CD8 mAb were incubated with Ts cells for 30 min in room temperature. Then cells were separated with a magnet according to manufacturer description and resulting CD8- and CD8+ cell populations were cultured separately for 48 h as described above. All supernatants were extracted with phenol chloroform via the procedure described below, and then used for standard treatment of adoptively transferred CS-effector cells.

### Transfer of CS-Effector Cells from TLR3^-/-^ Mice

Wild type (C57BL/6) and TLR3^-/-^ mice were contact sensitized with OX and 4 days later spleen and lymph node cells were harvested for treatment with progressively filtered supernatant from 48-hour OX-specific Ts cell culture with B1a cells. After incubation of 7 x 10^7^ cells with 3 ml of Ts supernatant (both amounts per recipient) for 30 min in 37°C water bath, the cells were washed with DPBS and adoptively transferred iv into naive wild type recipients, then ear challenged with 0.4% OX. After 24 h the intensity of the local CS reaction was assessed by ear swelling measurements as above.

### In Vitro Inhibition of HT-2 Cell Line Responsiveness to IL-2 by PCE and QRNA

The assay was performed as described previously [5,23] and the viability of HT-2 cells (ATCC, Manassas, VA) (10^4^ per well) was assessed after treatment with serially diluted PCE or QRNA samples at starting doses dependent on experiment protocol.

### Extraction of Supernatants with Phenol-Chloroform Mixture by Modified Chomczynski Method [24]

Culture supernatants were mixed at ratio 1:1 with phenol-chloroform mixture (5:1), buffered to pH = 4.7 (Sigma), extensively shaken for 5 min, and centrifuged at 4000g at 4°C for 15 min without brake. The upper water phase solution was recovered and the same phenol-chloroform extraction procedure was repeated. A third extraction was performed with phenol alone, equilibrated with 0.1M Tris-HCl buffer (pH = 8.0) and mixed with 0.2% 2-ME and 0.1% 8-hydroxyquinoline. This was followed by double extraction of the water phase solution with chloroform at a 3:1 ratio. The phases were separated by centrifugation at 4000g for 15 min at 4°C without brake. The separated pure aqueous fraction then was precipitated with 2M sodium acetate pH = 4.7 and ethanol at a volume ratio 10:1:33, respectively, followed by overnight incubation at -20°C. Then the precipitate was centrifuged for 35 min at 4000g, at 4°C. The pellet was washed twice with 70% ice cold ethanol, then dried and dissolved in pure water, and then measured in a spectrophotometer for estimation of DNA/RNA versus protein concentration; respectively at λ = 260 nm and 280 nm, and stored at -70°C. Such samples were called PCE (phenol-chloroform extract).

### Separation of PCE Samples on Qiagen Chromatography Columns to Obtain Highly Enriched RNA and DNA Fractions

Using a Qiagen RNA/DNA Maxi Kit, PCE fractions were separated into RNA (QRNA) and DNA (QDNA) fractions according to the manufacturer directions. The concentrations of obtained QRNA and QDNA were estimated spectrophotometrically at λ max 260 nm and λ max 280.

### Enzymatic Treatment of Ts Cell Supernatant-Derived PCE or Qiagen Column Fractions

PCE samples were treated with DNase at a dose of 1μg per 5μg of PCE for 30 min at 37°C in Tris-HCl buffer pH = 8.0. After incubation, the DNase-treated PCE was re-extracted with phenol chloroform, and precipitated with glycogen in ethanol mixture.

Ts Sup PCE samples in 20 ml of DPBS were treated with either RNase A (Sigma R-4375, 60% purity or Sigma R-5250, 95% purity) at a ratio of 333 μg per 1 mg of PCE, or with trypsin at a dose of 1 mg per 10 mg of PCE in analogous conditions as in DNase treatment, also followed by re-extraction with phenol chloroform to concentrate the sample and remove enzyme.

Treatment of the QRNA fractions with RNase III was performed according to the manufacturer at a dose of 1μg RNase III per 1μg of QRNA for 20 min at 37°C followed by phenol chloroform extraction and ethanol glycogen precipitation to remove the RNase III. QRNA fractions were also treated with Proteinase K at a dose of 1μg per 1μg of QRNA, in the presence or absence of 1% SDS, for 30 min at 37°C in TRIS-HCl buffer (pH = 8.0), followed by re-extraction with phenol chloroform and precipitation with ethanol glycogen mixture.

### Agarose 3% Gel Electrophoresis

Each sample of QRNA and QDNA was applied at a final concentration of 1μg per 10 μl of TBE (Tris-borate-EDTA buffer pH 8.0) per lane to a 3% agarose gel containing 0.002% ethidium bromide for electrophoresis lasting for 70 min at 45 mA and 100V, in comparison to non-separated PCE and low molecular DNA/RNA markers. Electrophoretically separated bands were visualized under UV light.

### Electrophoretic Separation of QRNA Fractions on 7M Urea, 12% Polyacrylamide Sizing Gel

Preliminary ligated with ^32^P-pCp Ts and Nl Cell Sup-derived QRNA (“hot” samples) [25] were used in trace amounts to obtain markers for subsequent sizing separation of preparative amounts of identical but unlabeled “cold” samples. Both, “hot” and “cold” samples (50μg per lane in TBE buffer containing 7M urea) were applied on 12% polyacrylamide sequencing gel in 7M urea. After electrophoresis lasting for 240 min at 50 mA and 100V, each “cold” sample band was eluted from the gel, precipitated with ethanol glycogen, then spectrophotometrically evaluated for RNA concentration, and then used to treat CS-effector cells prior to adoptive transfer and HT-2 cells in vitro.

### Blocking the Biological Activity of miRNA Present in QRNA Samples with Anti-miRs

A relevant set of anti-miRNA antagonists of miRNAs present in the QRNA samples [5] were used for blocking the suppressive activity of this fraction (Dharmacon RNAi Technologies). In each case, 3μg of appropriate anti-miR (165 pM for anti-miR to miR-150) was incubated with 3μg of Ts Sup-derived QRNA for 1 h at room temperature, followed by incubation of the mixture with TNP-CS-effector lymphocytes (7x10^7^ cells per recipient) for 30 min in 37°C water-bath. Then cells were washed at 300g with DPBS to remove the polynucleotides and then used for iv adoptive transfer into naive recipients, as above.

### Supplementation of Exosomes from 2 Day Contact Sensitized Mice with QRNA from Hapten-Specific Ts or Nl Cell Sup, or with Synthetic miR-150

Donors of the 2 day TNP-specific exosomes were contact sensitized with TNP-Cl as above. At day 2, spleen and lymph node cells were harvested and cultured (2 x 10^7^ cells per ml) for 48 h under the same conditions as Ts cells (see above). Exosomes from the Sup were obtained by preliminary centrifugations to clear debris and cells, progressive ultrafiltrations, and then ultracentrifugations (100,000g) for 70 min in DPBS [5,8]. The 100,000g pelleted exosomes were incubated with QRNA from Sup of either TNP Ts or Normal Cells (3μg QRNA per 8x10^9^ exosomes per eventual recipient; in a total volume of 300μL), and also in other groups with decreasing doses of miR-150 [Dharmacon]; starting with a dose of 3μg (equals 212 pM) of miR-150, again in 300μL. After incubation with appropriate RNA for 1h in a 37°C water bath, followed by washing with DPBS via the second ultracentrifugation at 100,000g for 70 min at 4°C, the RNA supplemented exosomes were used to treat TNP-CS-effector cells (8 x 10^9^ treated exosomes per 7x10^7^ cells per recipient, as assessed by NTA) for 30 min at 37°C. After washing with DPBS, the supplemented exosome-pulsed CS-effector cells were washed and then used in iv adoptive transfer of CS, as described above.

### Statistical analysis

All experiments were carried out two to four times. Double-tailed Student’s T-test as well as analysis of variance (ANOVA) with post-hoc RIR Tukey test were used to assess the significance of differences between groups, with p<0.05 taken as minimum level of significance.

## Results

### Phenol Chloroform Extraction Destroys Exosomes in the T Suppressor Cell Supernatant, but Remaining Extract (PCE) Is Still Suppressive

**Fig 1:**
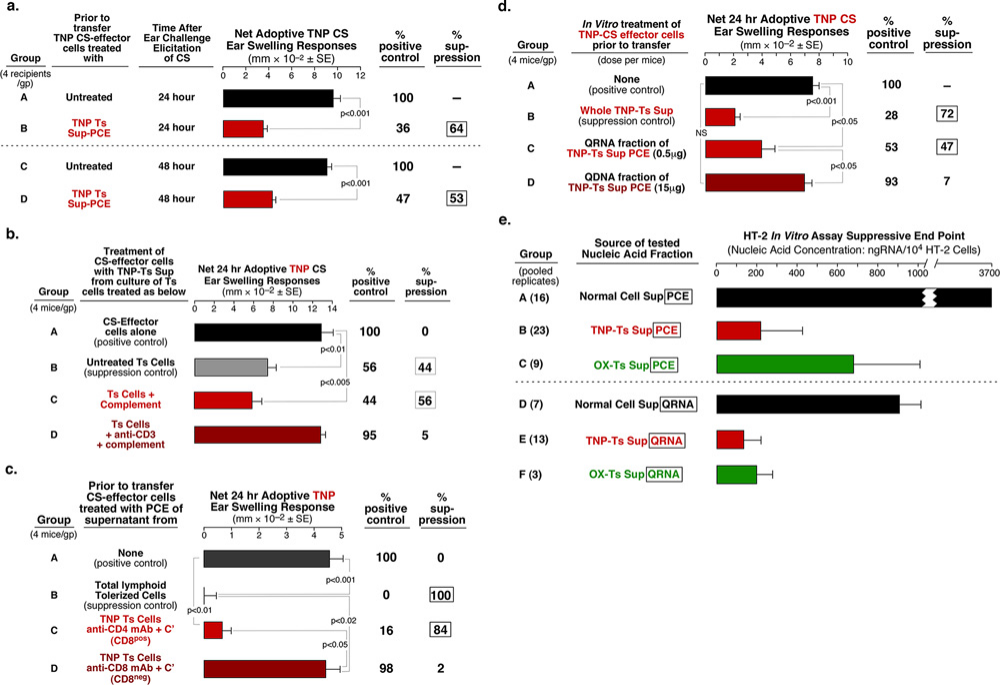
RNA from Phenol-Chloroform Extract (PCE) and Qiagen column fraction (QRNA) from CD3+ CD8+ suppressor T lymphocyte (Ts) culture supernatant (Sup) is suppressive. **a** **.** PCE from TNP Ts Sup suppresses the adoptive transfer of CS-effector cells (Groups B and D). **b**. Treatment of Ts cells with anti-CD3 mAb plus complement (C’) eliminates production of suppressive Sup (Group D). **c** **.** Similar treatment of Ts cells with anti-CD8 mAb plus C’ also eliminates generation of suppressive Sup (Group D), while anti-CD4 mAb plus C’ treatment does not (Group C). **d** **.** QRNA from TNP Ts Sup PCE separated on a Qiagen column suppresses adoptively transferred CS, while the QDNA fraction is inactive (Group C vs D). **e** **.** TNP- and OX-specific Ts Sup-derived PCE (Groups B and C) and QRNA (Groups E and F) inhibit HT-2 cell in vitro responsiveness to IL-2, compared to Nl Cell Sup PCE and QRNA (Groups A and D).

We tested if the exosomes from the Ts cell Supernatant (Sup) were required for the delivery of functional exRNA cargo (miR-150) to suppress CS. The exosomes were completely destroyed during the phenol/chloroform extraction, as shown by nanoparticle tracking analysis [5]. Unexpectedly, the remaining solubilized nucleic acid-enriched PCE still suppressed adoptively transferred CS-effector cell responses induced by trinitrophenyl (TNP) immunization (Fig 1a, group A vs B and C vs D). Similarly, PCE administered ip just after challenge inhibited T cell-dependent 24 h CS response (S1 Fig, group C vs D), while early phase 2 h ear swelling reaction dependent on B1a cells [6,7] was unaffected (S1 Fig, group A vs B). This allowed us to exclude non-specific cytotoxicity of PCE treatment on the transferred cells. Similarly, PCE from the Ts Sup of mice tolerized with another hapten (oxazolone, OX) also suppressed OX-induced CS (S4 Fig, group B vs A). These results led to a series of experiments summarized in S9 Fig to characterize the entity in the PCE containing mixed nucleic acids that surprisingly mediated suppression in the absence of exosomes.

### Suppressive PCE Is from CD3+ CD8+ Suppressor T Cells

Depletion of CD3+ cells with anti-CD3 mAb plus complement before culture, eliminated suppression mediated by the Ts Sup (Fig 1b, group D vs B and C). Further, removal of CD8+ cells with anti-CD8 mAb plus complement prior to cell culture, resulted in Sup that after extraction to PCE was non-inhibitory (Fig 1c, group D). As confirmation, removal of CD8+ cells from the tolerized lymphoid cells with Ab coated magnetic beads prior to culture gave similar results (S2 Fig, group D vs C). An analogous cell depletion effect was observed in tolerance to the non-cross reacting OX hapten [5]. In contrast, CD4+ cell removal did not affect suppression mediated by PCE derived from Ts Sup (Fig 1c, group C). We therefore concluded that suppressive part of Ts Sup present in PCE was derived from CD3+ CD8+ T cells.

### Suppression Is Mediated by the RNA Fraction from Exosome-Free PCE

**Fig 2:**
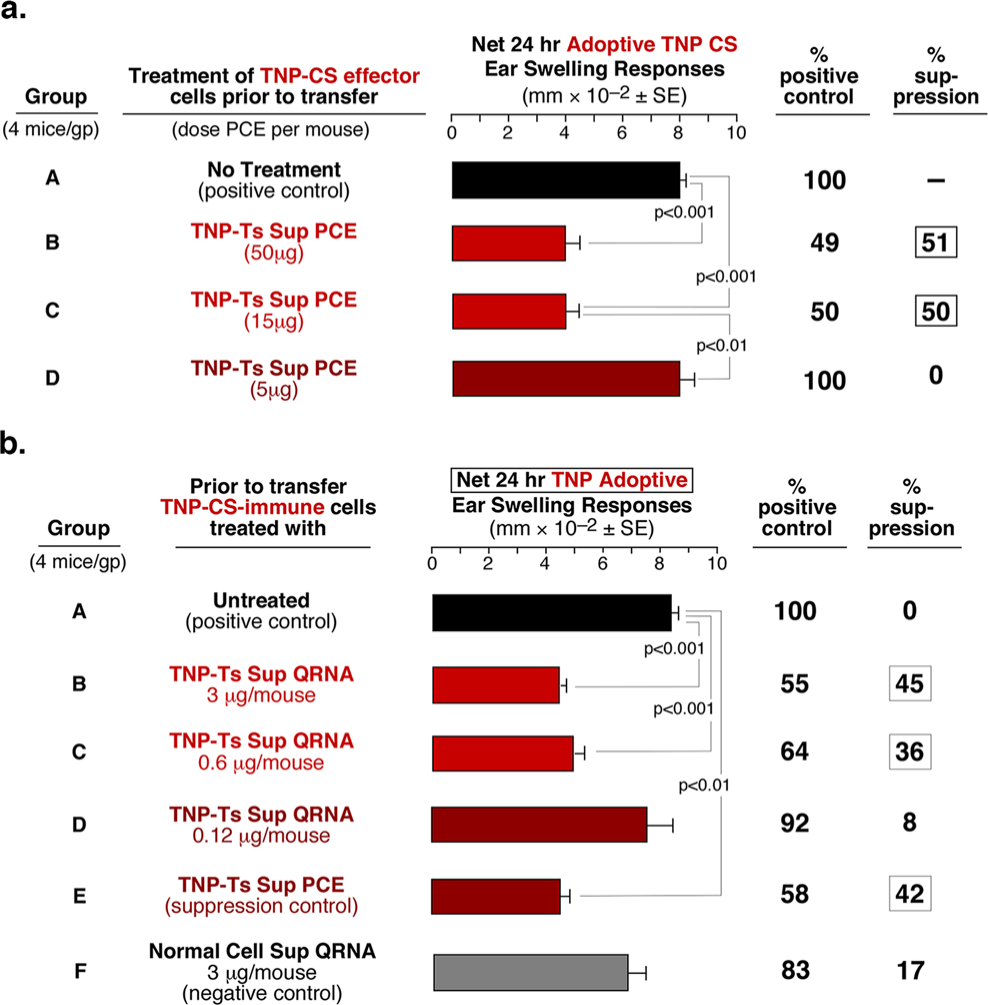
Dose-dependent suppression mediated by PCE and QRNA fractions of TNP Ts Sup. **a**. Three-fold decreasing doses of TNP Ts Sup-derived PCE regressively suppresses adoptive CS responses down to a dose of 15μg per eventual recipient (Groups B and C). **b**. Five-fold decreasing dose-response of QRNA from TNP Ts Sup PCE results in suppression of adoptive CS responses down to a dose of 0.6μg per recipient (Group C).

Using Qiagen RNA/DNA separating columns we determined that the RNA fraction (QRNA), that was only 5% of the PCE, suppressed adoptively transferred CS-effector cells at a dose of 0.5μg per individual recipient, whereas the DNA fraction was not suppressive, even at a 30 fold greater dose (Fig 1d, group C vs D), nor was the QRNA fraction of cell culture supernatant from normal non-immune mice (Nl Cell Sup) (Fig 2b group F).

Since CS reactions depend on various cytokines, including IL-2 as a potent inductor of T cell proliferation, we assessed the influence of the tested suppressive fractions on IL-2 activity. We showed previously that the *in vitro* responsiveness of the HT-2 T cell line to IL-2 [23] was inhibited by the suppressive exosomes [5]. The exosome free TNP Ts Sup PCE or QRNA had similar suppressive activity (Fig 1e, group B and E). Additionally, exosome free OX Ts Sup PCE and QRNA from mice tolerized with OX also were suppressive in this Ag-non-specific in vitro assay (Fig 1e, group C and F), while PCE and QRNA from Nl Cell Sup, even at high doses, were non-inhibitory (Fig 1e, group A and D).

### Suppression by exRNA without Exosomes Is Dose Dependent

Three fold decreasing doses of Ts Sup PCE from mice tolerized with TNP regressively inhibited CS-effector cell adoptive transfer down to a dose of 15μg per recipient (Fig 2a, group C). Further, dose-dependent treatment with the QRNA showed that as little as 0.6μg was the limiting suppressive dose (Fig 2b, group C). Similarly, dose-dependent treatment with the Ts Sup PCE and QRNA inhibited the in vitro HT-2 cell response to IL-2 down to doses of only 360ng and 74ng, respectively. Analogous results were obtained with the total RNA in the PCE and QRNA from Ts Sup of mice tolerized with OX (S3a and S3b Fig).

### Enzyme Treatments Show That Double Stranded exRNA Is Responsible for Suppression

**Fig 3:**
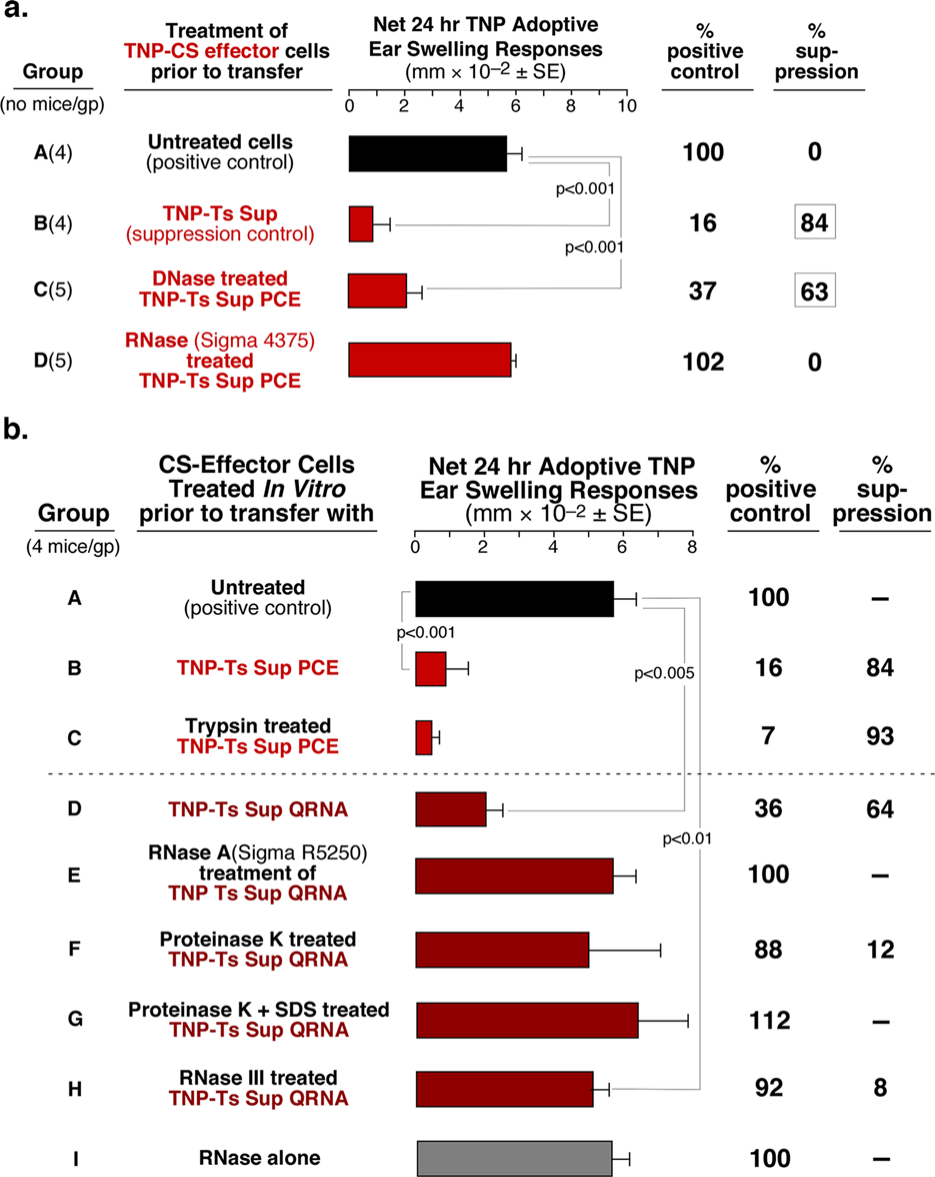
Enzymatic treatment of nucleic acids and associated proteins from PCE and QRNA of TNP Ts Sup eliminates suppressive activity. **a**. TNP Ts Sup-derived PCE suppression is sensitive to RNase A (Sigma 4375), but not DNase treatment (Group D vs C). **b**. Purer RNase A (Sigma 5250, Group E) and RNase III (Group H), as well as proteinase K with and without SDS (Groups F and G) treatment of QRNA from Sup of TNP Ts, eliminates its suppressive activity.

To confirm involvement of RNA, the PCE and QRNA of TNP Ts Sup were enzymatically treated with RNase or DNase. RNase A (Sigma 4375) treatment eliminated suppression mediated by the PCE fraction of TNP Ts Sup (Fig 3a, group D), as well as by OX Ts Sup PCE (S4 Fig, group C). In contrast, there was no inactivating effect of DNase treatment (Fig 3a, group C). Further, highly purified RNase A (Sigma 5250), used to eliminate possible contamination of the previously employed RNase (Sigma 4375) with other enzymes, also significantly impaired TNP Ts Sup QRNA activity (Fig 3b, group E). Importantly, RNase III, specific for double-stranded RNA (dsRNA), also eliminated the QRNA-mediated suppression (Fig 3b, group H). It is noteworthy that after each RNase treatment, the remaining nucleic acid mixture was re-extracted with phenol chloroform to remove any contaminating enzymatic protein that might influence the subsequent CS-effector cell transfers. In addition, treatment with RNase alone did not suppress the adoptively transferred CS response (Fig 3b, group I). Agarose gel electrophoretic analysis comparing the resulting TNP and OX PCE and QRNA bands to commercial DNA and RNA standards, confirmed that the enzyme treatments were successful; i.e. RNase removed RNA and not DNA, and DNase removed DNA and not RNA from the PCE and QRNA. Thus suppression mediated by the mixed nucleic acids in the PCE and by QRNA was due to an extracellular dsRNA acting in the absence of exosomes.

### Ts Sup exRNA May Be Protected from RNases by an Associated Protein

Effects of proteolytic enzyme treatment on the suppressive free exRNA was tested, since exRNA often are bound to associated protective protein chaperones, like the Argonaute family [26–28] or lipoproteins [29,30]. Treatment of the Ts Sup PCE with trypsin had no effect on its activity (Fig 3b, group C), whereas more specific treatment of the enriched QRNA with proteinase K reduced, and in the presence of sodium dodecyl sulfate (SDS) eliminated suppression (Fig 3b, group F and G). Thus, a protective hydrophobic protein was likely bound to the free exRNA.

### Suppression Was Not Due To TLR3 Activation by the dsRNA of Isolated exRNA

**Fig 4:**
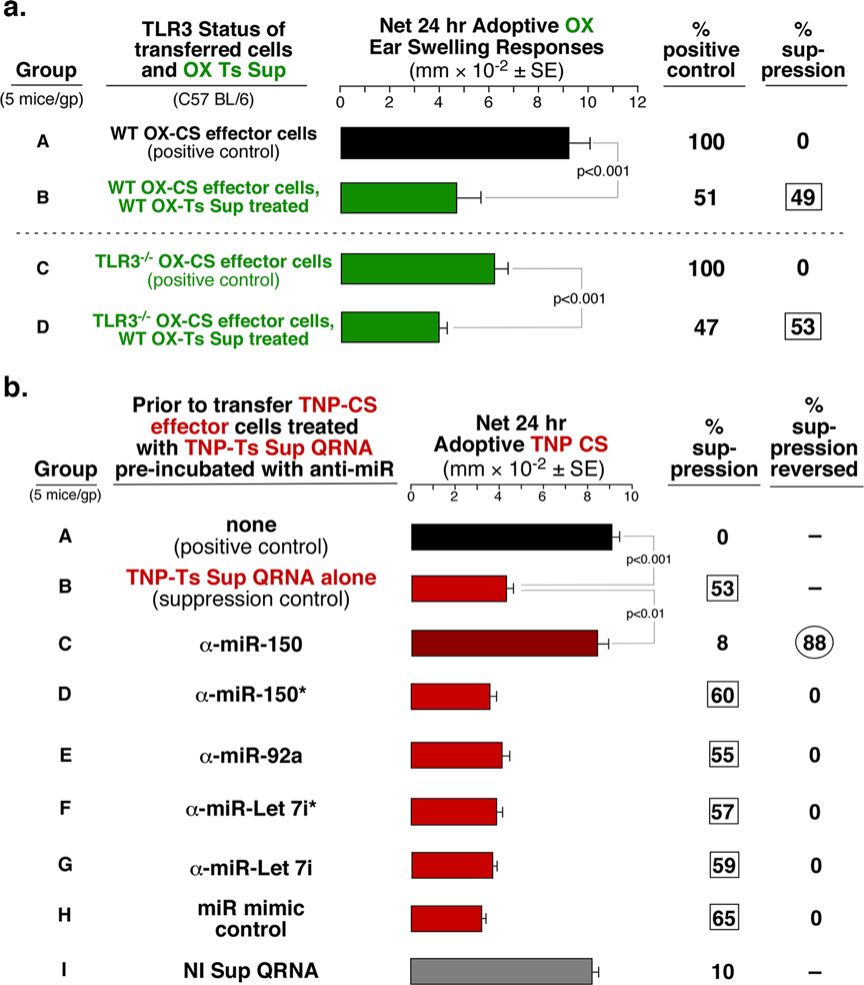
Suppression mediated by dsRNA is not TLR3-dependent and can be blocked by anti-miR to miR-150. **a**. Treatment of CS-effector cells from either wild type (WT) or TLR3^-/-^ mice with OX Ts Sup of WT mice results in equal suppression of the CS immune response (Group B and D). **b** **.** Among different miRNA antagonists (groups C-G), only anti-miR to miR-150 is able to reverse the suppression of CS mediated by TNP Ts Sup QRNA (group C).

To determine if the TLR3 signaling pathway activated by the dsRNA was involved in the suppression mediated by free QRNA, C57BL/6 wild type (WT) and TLR3^-/-^ mice were contact sensitized with OX hapten. Adoptive transfer of CS by harvested CS-effector cells from either WT or TLR3^-/-^ mice was similarly suppressed by Ts Sup from WT mice (Fig 4a, group B and D). Therefore, dsRNA in the PCE or QRNA did not act via TLR3 to suppress the transferred CS-effector cells, although other dsRNA sensing pathways might be involved.

### Suppression Was Due To a Small exRNA of About 75bp

Polyacrylamide gel electrophoretic sizing was performed. QRNA from TNP Ts Sup and Nl Cell Sup were labeled with P^32^ to obtain analytic markers (S5a Fig). They were then used to indicate sizes of electrophoretically separated unlabeled 100μg QRNA preparative fractions of Ts Sup (S5b Fig). These unlabeled fractions were eluted from the separating gel and tested for suppression of CS-effector cell adoptive transfer and HT-2 cell responsiveness to IL-2. These assays showed that fractions of about 75 base pairs from the QRNA of TNP Ts Sup were inhibitory when compared to those from Normal Cell QRNA, and were suppressive in vitro in a dose of 3–4ng of total RNA (S6a Fig group D and E; S6b Fig group B and C). Altogether, the data showed that suppression was mediated by small amounts of short extracellular dsRNA free of exosomes; possibly pre-miRNA or miRNA.

### Anti-miRNA To miR-150 Blocks Suppression Mediated by the Ts Sup Total QRNA

The free QRNA from Ts Sup PCE functioned similarly to the previously described Ts Sup exosomes that suppressed the CS-effector cell mixture due to their content and delivery of miR-150 [5]. Since this was determined in part by anti-miR blockage, we tested the same set of relevant anti-miRNAs [5] for reversal of CS-effector cell suppression by free QRNA. Remarkably, only anti-miR to miR-150 reversed suppression of CS adoptive cell transfer (Fig 4b, group C vs B). This suggested that miR-150 could be crucial among the mixture of exRNAs, as it is for the Ts Sup exosome mediated suppression [5]. However, it remained unclear how this inhibitory miRNA played its role as free RNA, devoid of exosomes.

### Suppression Mediated by the exRNA Seems To Be Ag-Specific

**Fig 5:**
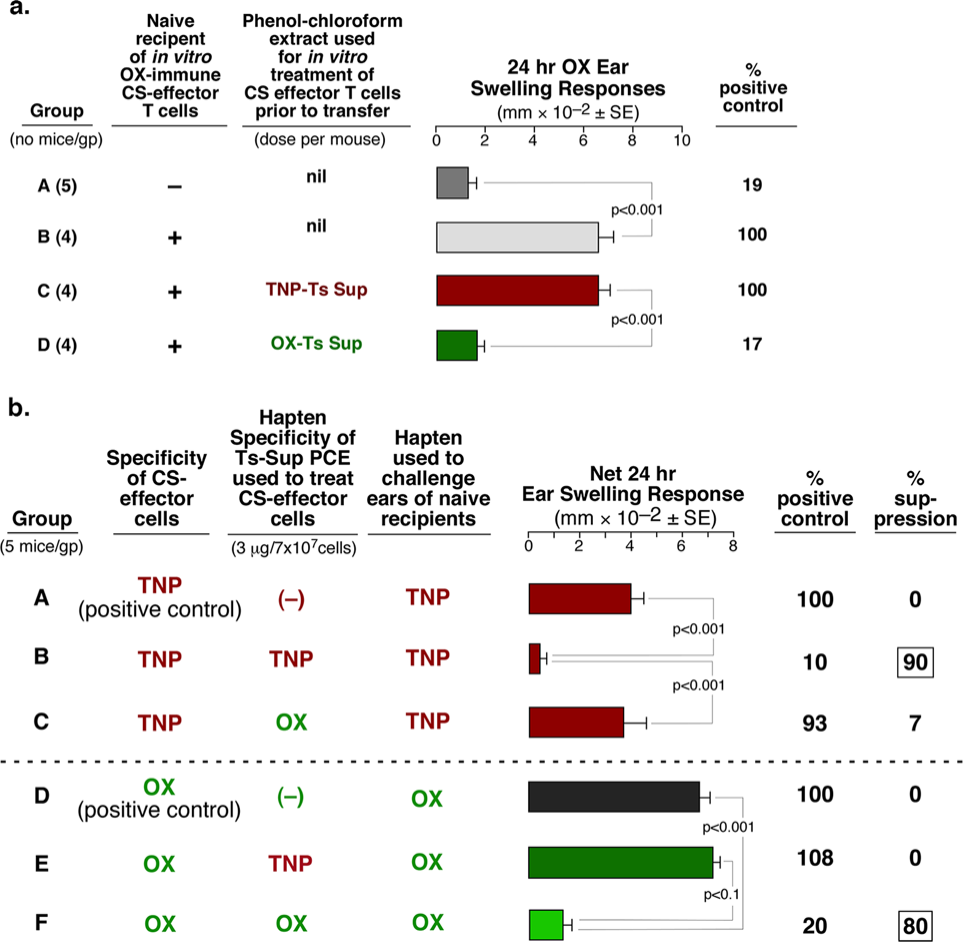
Possible Ag-specific suppression by Ts Sup exRNA PCE. **a** **.** OX specific CS-effector cells were suppressed by OX Ts Sup PCE, but not TNP Ts Sup PCE (Group D vs C). **b**. In a dual reciprocal criss cross Ag-specificity experiment, TNP vs OX-specific CS-effector cells were suppressed by PCE from Ts Sup induced by tolerization with their respective homologous TNP or OX haptens (Groups B and F), but not from mice tolerized with the heterologous hapten (Groups C and E).

We surprisingly found that PCE of OX Ts Sup suppressed transfer of OX CS-effector cells, but not TNP CS-effector cells (Fig 5a group C vs B). To further test the hypothesis that free exRNA released from exosomes by phenol chloroform extraction may suppress CS-effector cells in homologous Ag-specific manner, a dual reciprocal criss cross Ag-specificity experiment was performed. PCE of TNP Ts Sup strongly suppressed TNP CS-effector cell adoptive transfer (Fig 5b group B), but not OX CS-effector cells (Fig 5b group E). In contrast, OX Ts Sup PCE strongly suppressed OX CS-effector cells (Fig 5b group F), but not those transferring TNP-induced CS (Fig 5b group C vs B). Similarly, in QRNA-mediated suppression, the activity was more effective in the hapten homologous system than in the heterologous system (S7 Fig). These findings suggested that free exRNA released from Ag-specific exosomes may act efficiently only in Ag homologous system leading to Ag-specific suppression.

### Neither Immunoglobulins nor Ag-Binding Capacity Explain Ag-Specific Suppression by Free exRNA

Repeated mass spectroscopy analysis of free exRNA performed in the Institute of Biochemistry and Biophysics of Polish Academy of Sciences failed to show any immunoglobulins in PCE or QRNA samples. Further, S8 Fig shows almost identical transcriptomes of QRNA fractions before and after Ag-affinity chromatography enrichment [5] (Sample No 3 and 4), similar to unfractionated Ts Sup and Nl Cell control QRNA (Sample No 1 and 2). In contrast, Ag-affinity fractionation of the Ag-binding suppressive exosomes vs the Ag-non-binding exosomes in the suppressive TNP Ts Sup exosome mixture [5] resulted in different transcriptomes (Sample 5 and 6). Thus, Ts Sup QRNA expressed no immunoglobulins, and it was not fractionated by Ag-affinity chromatography, but the contained exRNAs inhibited transferred CS in a homologous Ag-specific manner (Figs 5 and S7). We concluded that the antigen-specific activity of free exRNA was not based on direct content of antibodies, in contrast to the suppressive T cell exosomes [5]. Thus, we sought other mechanisms for the seeming Ag-specificity of free exRNA.

### exRNA Association with Exosomes from B1a Cells in the CS-Effector Cell Mixture May Provide Its Ag-Specific Activity

We previously showed that incubation of non-suppressive exosomes from tolerized miR-150^-/-^ mice with synthetic miRNA-150 rendered them suppressive [5]. Similarly, incubation with miR-150 antagonist blocked suppression mediated by miR-150 containing exosomes from tolerized wild type mice [5]. We postulated that these observed effects of free exRNA association with exosomes may be due to miR-150 binding to exosome surface lipids, as recently shown for Ab LC [31], and possibly active influx via SID-1 channel [32], that we herein termed transfection. Further, in other prior work on the initiation of elicitation of T cell-mediated CS, we showed that the adoptively transferred CS-effector cell mixture, used here to assay free exRNA function, contained a subpopulation of very early activated B1a cells [6,7]. These cells produce antibodies rendered Ag-specific via activation induced deaminase (AID)-dependent mutations in the immunoglobulin heavy chain variable region [6]. B1a cells are required in CS early phase for production of Ag-specific IgM and Ab LC that initiate local recruitment of the CS-effector cells via complement and mast cells activation, respectively [6,7]. Therefore, we postulated that these B1a cells present in the assayed CS-effector cell mixture might produce exosomes coated with appropriate Ag-specific B cell receptors (BCR) or Ab LC. In that case, it would be possible that free exRNA could interact with these B1a cell-derived exosomes to deliver the actual inhibitory entity: miR-150, and Ag-specifically suppress companion CS-effector T cells.

**Fig 6:**
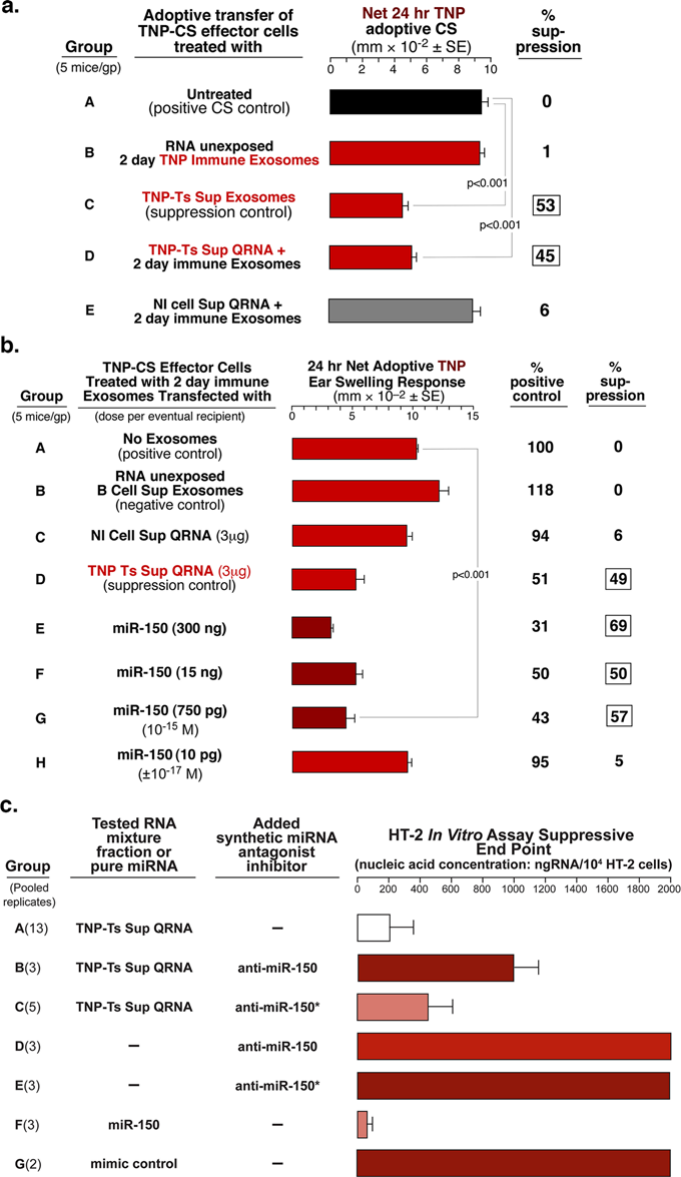
Suppression by extracellular QRNA from TNP Ts Sup free of exosomes, or miR-150 alone, associating with B-cell derived Ag-specific exosomes from the assayed CS-effector cell mixture. **a.** Treatment of TNP-CS-effector cells with two day immune B-1 B cell-derived TNP-specific exosomes that are supplemented with QRNA from TNP Ts Sup (Group D) results in significant suppression of the adoptive transfer. **b.** Similarly, two day immune B-1 B cell-derived exosomes, supplemented with decreasing doses of miR-150 alone, mediated suppression of TNP-CS-effector cell adoptive transfer (Groups E-G), down to a dose of 750pg per eventual recipient, which is 50 femtomoles per eventual recipient (Group G). **c.** The in vitro HT-2 cell responsiveness to IL-2 was suppressed by QRNA from Ts Sup and this was inhibited by pre-incubation of the QRNA with anti-miRNA-150, but not by anti-miRNA-150* (Groups B and C). Further, pure miR-150 alone was inhibitory compared to mimic control (Groups F vs G).

To test this hypothesis, we contact sensitized naive mice with TNP-Cl and 2 days later harvested spleens and lymph nodes. We previously showed that this was optimal method for obtaining early activated Ag-specific B1a cells [6,7]. After 48 h culture their supernatant was processed for exosomes [5], expected to have a possible Ag-specific surface coating. The pelleted putative Ag-specific exosomes were incubated with QRNA of Ts Sup from TNP tolerized mice, and then washed by 100,000g ultracentrifugation to remove free RNA. Interestingly, these putative B1a cell-derived Ag-specific exosomes, that alone were not suppressive (Fig 6a group B), when pulsed with TNP Ts Sup QRNA and washed, became suppressive, similarly to our standard TNP Ts Sup-derived T cell exosomes from TNP tolerized mice [5] (Fig 6a, group D vs C). In contrast, 2-day TNP-Cl induced exosomes supplemented with control Nl Cell Sup QRNA were not suppressive (Fig 6a, group E). We concluded that the association of exRNA with non-suppressive B1a cell-derived Ag-specific exosomes from the effector cell mixture, that was used for adoptive CS transfer, enabled Ag-specific inhibition of companion CS-effector T cells. The exact characteristics of these B1a cell exosomes, in comparison to the T cell exosomes from the tolerized mice, as well as their putative selective transfection with exRNA, are the subject of ongoing studies.

### Ag-Specific B1a Cell Exosomes Mediate Suppression When Supplemented with Pure miR-150 in Minute Amounts

In a follow-up experiment, the 2-day TNP-Cl exosomes were supplemented with miR-150 alone, and mediated suppression of CS-effector cell adoptive transfer, similarly to QRNA of TNP Ts Sup (Fig 6b, group E and D vs A). This finding was consistent with the results of the experiment with anti-miR to miRNA-150 (Fig 4b) and confirmed that miR-150 is crucial for suppression mediated by Ts Sup QRNA. Further, a dose response experiment showed that the early Ag-specific B1a cell exosomes supplemented with as little as 750pg (50 femtomoles, 50 x 10^-15^) of pure miR-150 per recipient suppressed the adoptive transfer of CS-effector cells (Fig 6b, group G).

### Pure miR-150 Suppresses In Vitro Responsiveness of HT-2 Cells to IL-2 and Anti-miR-150 Reverses Suppression Mediated by QRNA

We aimed to determine if miR-150 might influence the HT-2 cells’ in vitro responsiveness to IL-2. We found that miR-150 alone completely suppressed HT-2 cell responsiveness to IL-2 when compared to mimic control (Fig 6c group F vs G). We also investigated whether anti-miR-150 blocked the observed in vitro suppression mediated by the Ts Sup-derived QRNA mixture, likely acting due to its miR-150 content. Indeed, the miR-150 antagonist blocked suppression of the HT-2 cell responsiveness (Fig 6c, group B vs A), while the antagonist of the reverse sequence carrier chain miR-150* was much less efficient (Fig 6c, group C vs B). We concluded that miR-150 was responsible for suppression mediated by exosome-free QRNA derived from Ts Sup observed both in vivo (Fig 6b) and in vitro (Fig 6c), which was confirmed with specific antagonists in vivo (Fig 4b, group C) and in vitro (Fig 6c, group B).

## Discussion

### Synopsis: A New Paradigm of Cell to Cell Transfer of Extracellular miRNA Free of Exosomes

We have shown that Ag-specific suppression of CS is mediated by free exRNA. This exRNA suppresses effector T cells and is present in the PCE and QRNA fractions of T suppressor cell supernatant. We have discovered that the mechanism of its suppression involves its eventual association or transfection of exosomes from B1a cells accompanying the assayed CS effector T cells, the final target of suppressive exRNA. The suppression is executed by miR-150 in the exRNA, the miRNA previously shown to be responsible for suppression mediated by CD8+ suppressor T cell-derived exosomes, that also were the source of the exRNA here. Induced during tolerogenesis suppressor T cell-derived exosomes transferred and protected from RNases the suppressive miR-150, as well as other RNAs. This protection was obliterated during the phenol-chloroform extraction, yet still the released free exRNA was suppressive in both adoptive transfer of CS-effector cells in vivo and in HT-2 cell line responsiveness to IL-2 in vitro. This exosome free PCE of exRNA remained suppressive when proceeded to Qiagen RNA, and further to polyacrylamide electrophoretic sized dsRNA fractions. Surprisingly, PCE and to a lesser extent QRNA acted in homologous antigen-specific manner. As noted, this enigmatic Ag-specificity of exRNA-mediated suppression led to unprecedented demonstration that free exRNA can act by association with exosomes produced by the companion cells.

The fact that the tested exRNA (and finally RNA-pulsed exosomes) act Ag-specifically led to the unraveling the mechanism of endocrine intercellular regulation by free exRNA. It is possible that exRNA that does not act Ag-specifically also might use interaction with exosomes from cells accompanying the actual target cells, or even the targeted cells themselves, as a mode of transfer and protection of genetic instructions.

### Generation of a System to Study an Alternate Pathway of Intercellular Communication by exRNA That Is Free of Exosomes

In the prior study we showed that Ts cell-derived exosomes via a surface coat of Ab LC Ag-specifically delivered miR-150 to suppress CS-effector cells [5]. Further, association of inhibitory miR-150 alone with Ab coated non-suppressive T cell exosomes from tolerized miR-150 deficient mice restored their suppressive activity [5]. The current study tested the applicability of this exosomal mechanism for carriage, targeting and delivery of free exRNA. Molecular biology techniques and enzymatic treatments demonstrated that the inhibitory activity of free exRNA is due to small dsRNA. Further, its suppression was blocked in vivo and in vitro by anti-miR-150 alone; suggesting that miR-150, representing a very small fraction of the total RNA in the PCE and QRNA, is responsible. Indeed, pure miR-150 supplementation rendered non-suppressive Ag-specific exosomes, likely derived from companion B1a cells, suppressive; even at such small amounts as femtomoles; here a nanomolar concentration (Fig 6b). Notably, TNP Ts Sup-derived QRNA separated on polyacrylamide electrophoresis sizing gels yielded 75bp fractions active in the in vitro HT-2 cell assay at a dose of about 4ng total RNA (S6b Fig). If 0.01% of this total QRNA fraction is miR-150 (perhaps a conservative estimate) the dose efficient for in vitro function of miR-150 in this mixed material is also in the picogram range; again femtomolar amounts. Together these findings show the sensitivity and significance of the described system, in which currently undetectable amounts of a specific miRNA have robust in vivo and in vitro biological effects. Therefore, our study may offer a model for a possible general physiological alternate pathway of transmission of exRNA free of exosomes to target and then influence acceptor cell function. Further, it provides a system for study of ordinarily undetectable amounts of miRNA assumed to be non-functional, but here proved to be bioactive in vivo and in vitro.

### Biological Relevance

Free exRNA represents a substantial proportion of RNA in the circulation [33–38]. It is receiving much attention as potential biomarker of various diseases [39,40]. However, there are no prior reports nor has it been mentioned in reviews [41–43] that free exRNA can transfer functional information to acceptor cells. In such circumstances an intermediate role of exosomes produced by the targeted acceptor or companion cells should be considered. In our system, Ag-specific Ab or BCR coat of such exRNA-associated exosomes from the assayed population allowed for Ag-specific targeting of cells. In conclusion, the studied system, that allowed us to unravel a mechanism of exRNA mediated intercellular regulation seemingly free of exosomes, needs three fundamental conditions: **a**. Ag-specific Ab or BCR, **b**. its presence on the surface of exosomes **c**. that carry miR-150. It is interesting that in this system the associable exosomes might come from either T [5] or B cells.

### Ag-Specificity of Suppression Mediated by Exosome Free Ts Sup exRNA Acting Via B Cell Exosomes

The dual reciprocal criss cross Ag-specificity experiments employing the PCE or QRNA as free exRNAs, showed that they acted Ag-specifically. As mentioned, the suppressed assayed CS-effector cell mixture used for the assay contained a subpopulation of Ag-specific B1a cells we had described previously [6,7]. They are activated early after CS-immunization and are required for eliciting of transferred and active CS [6,7] and Delayed Type Hypersensitivity (DTH) responses [3]. They act in an early effector molecular and cellular initiating cascade leading to local recruitment of the effector T cells into the tissues [6,7]. These B1a cells produce Ag-specific Ab LC [12,13] and IgM [7,13] of sufficient affinity due to Ig heavy chain variable region mutations dependent on AID [6]. Interestingly, the current data suggests that such B1a cells also may produce exosomes coated with Ab or BCR for association with inhibitory free exRNA and even isolated miR-150 to become suppressive. Thus, we suggest that the same subpopulation of early activated Ag-specific B1a cells may act in both the effector and suppressive pathways.

### Classical vs Alternate Intercellular Modes of exRNA Exchange

The classical route of intercellular transit of free miRNA to target cells is mediated by exosomes from the terminal endosome of the donor cell—multivesicular body of the terminal endosomal pathway (MVB), as defined by us previously [5]. In the alternate pathway proposed here, miRNA is released by donor cells as free exRNA, interacts with exosomes from the target acceptor or companion cells and is uptaken and then transfered to target cells, where it performs its function. The finding that free exRNA after lysing the endocrine-acting exosomes had similar suppressive activity as miRNA in the exosomes, generated a model system to study intercellular regulation via free miRNA.

The alternate pathway of exRNA cargo delivery to acceptor cells described here likely occurs naturally and is important since significant amounts of RNA in the circulating blood are not carried by vesicles [33–38]. Published data claims that the free exRNA is protected from RNases by a hydrophobic protein chaperone like Argonautes [26–28] that might participate in miRNA transit between cells, Our preliminary experiments have shown that the suppressive activity of free exRNA can be blocked with anti-Argonaute Ab [32] suggesting an active role of Argonaute in the transit of free exRNA. To our knowledge, the alternate pathway we uncovered is the first demonstration that freely circulating miRNA can inhibit the function of acceptor cells, and dose dependently associate with exosomes produced by the target cell population. This appears to be the only natural mechanism yet described for passing functional free extracellular miRNA between cells.

### Translational Implications of the Current Studies

There has been a major effort put into developing siRNA therapy by its delivery in artificial carrier molecules or nanoparticles for silencing mRNA of particular genes. However, the artificial carriers are perceived by the body as foreign. This often leads to their rapid removal by the reticuloendothelial system, catabolism in the liver, and discharge in the urine. Exosomes are a more physiological carrier to deliver therapeutic miRNA or siRNA. Importantly, and thus far uniquely demonstrated here in the alternate pathway, and previously in the classical pathway of miRNA cell transfer via exosomes [5], the miRNA can be delivered Ag-specifically. This is a yet another important property not achieved by any artificial nanoparticles. This pathway is likely of great significance for delivering miRNA with highly antigen specific activity, long physiological duration, far fewer side effects, and ability to cross the blood brain barrier [4,44,45]. Altogether, discovery of two pathways of miRNA cell transfer is a result of reinvestigation of formerly characterized immunosuppression mechanism mediated by hapten-specific T suppressor factor [46].

In conclusion, delineation of two pathways for exRNA Ag-specific targeting, and association of selected miRNA with exosomes, allowing for subsequent exquisitely specific induction of chosen genetic alterations of acceptor cell functions, should further encourage examination of exosomes as a natural and very effective therapeutic platform for RNAi based therapeutics.

## Supporting Information

S1 Fig Treatment of actively sensitized mice with PCE of TNP Ts Sup preferentially suppresses the late component of CS. Treatment of actively TNP contact sensitized mice with PCE from TNP Ts Sup results in suppression of the classical 24h component of elicited CS, but not the early 2h CS component (Group D vs B).
(TIF)

S2 Fig CD8+ T lymphocytes (Ts) produce suppressive supernatant (Ts Sup). Only PCE from the fraction of Ts Sup isolated from magnetic bead separated CD8+ cells from TNP tolerized mice suppresses TNP-CS-effector cell transfer (Group C vs D).
(TIF)

S3 Fig Suppression mediated by PCE and QRNA fractions of OX Ts Sup is dose-dependent. **a.** Decreasing doses of PCE from OX Ts Sup regressively suppress adoptive CS responses down to a dose of 11μg per recipient (Groups B to D). **b.** Similarly, dose-response treatment with QRNA from OX Ts Sup showed that QRNA suppresses CS response down to a dose of 1μg per recipient (Group C).
(TIF)

S4 Fig Suppression of CS mediated by OX Ts Sup PCE is sensitive to RNase treatment. Treatment with RNase of PCE from OX Ts Sup eliminates suppression of adoptively transferred CS response (Group C vs B).
(TIF)

S5 Fig Electrophoretic separation of suppressive exRNA. **a** **.** Radioautographic visualization of electrophoretically separated fractions of P^32^ labeled QRNA of TNP Ts Sup on a 12% polyacrylamide sizing gel. **b** **.** Preparative fractions of unlabeled QRNA from TNP Ts and Nl Cell Sup to test in adoptive transfer of CS in vivo and inhibition of HT-2 cell responsiveness to IL-2 in vitro.
(TIF)

S6 Fig Approximately 75bp sized fractions of QRNA from TNP Ts Sup inhibit CS in vivo and HT-2 responsiveness to IL-2 in vitro. **a**. Only RNA fractions of about 75bp from TNP Ts Sup QRNA separated on sizing gel inhibit adoptively transferred TNP-CS-effector cells (Groups D and E) compared to unseparated TNP Ts Sup QRNA (Group B), whereas Nl Cell Sup QRNA fractions were not suppressive (Groups H and I). **b**. Similarly, TNP Ts Sup-derived QRNA (about 75bp) fractions separated on sizing gel inhibit IL-2 dependent viability of HT-2 cells (Groups B and C), while related fractions of Nl Cell Sup were not suppressive (Groups F and G).
(TIF)

S7 Fig Ag specificity of QRNA suppression of CS-effector cell adoptive transfer of CS. TNP vs OX-specific CS-effector cells were strongly suppressed by QRNA from Ts cell induced by respective homologous hapten (Groups B and F), whereas QRNA from heterologous hapten induced Ts cells was significantly less effective (Groups C and E).
(TIF)

S8 Fig Comparison of transcriptomes of TNP Ts Sup QRNA and TNP Ts Sup exosomes demonstrating the absence of Ag-binding by QRNA. QRNA from TNP Ts Sup before and after separation with TNP-affinity column chromatography express almost identical annotations of RNA subtypes and thus very similar transcriptomes (samples 3 and 4), comparable to QRNA from Nl Cell Sup (Sample 2), whereas TNP-affinity separation of exosomes from TNP Ts Sup fractionates exosomes into Ag-binding (suppressive) and non-binding (non-suppressive) fractions with very different transcriptomes (Sample 5 vs 6).
(TIF)

S9 Fig Experimental design. Figure shows the scheme of further preparation of Ts Sup and experimental usage of resulting fractions.
(TIF)
