# Supplementary figures and images for: Free Extracellular miRNA Functionally Targets Cells by Transfecting Exosomes from Their Companion Cells
Source: PLoS One. 2015 Apr 29;10(4):e0122991. doi: 10.1371/journal.pone.0122991 (PMC4414541; doi:10.1371/journal.pone.0122991)

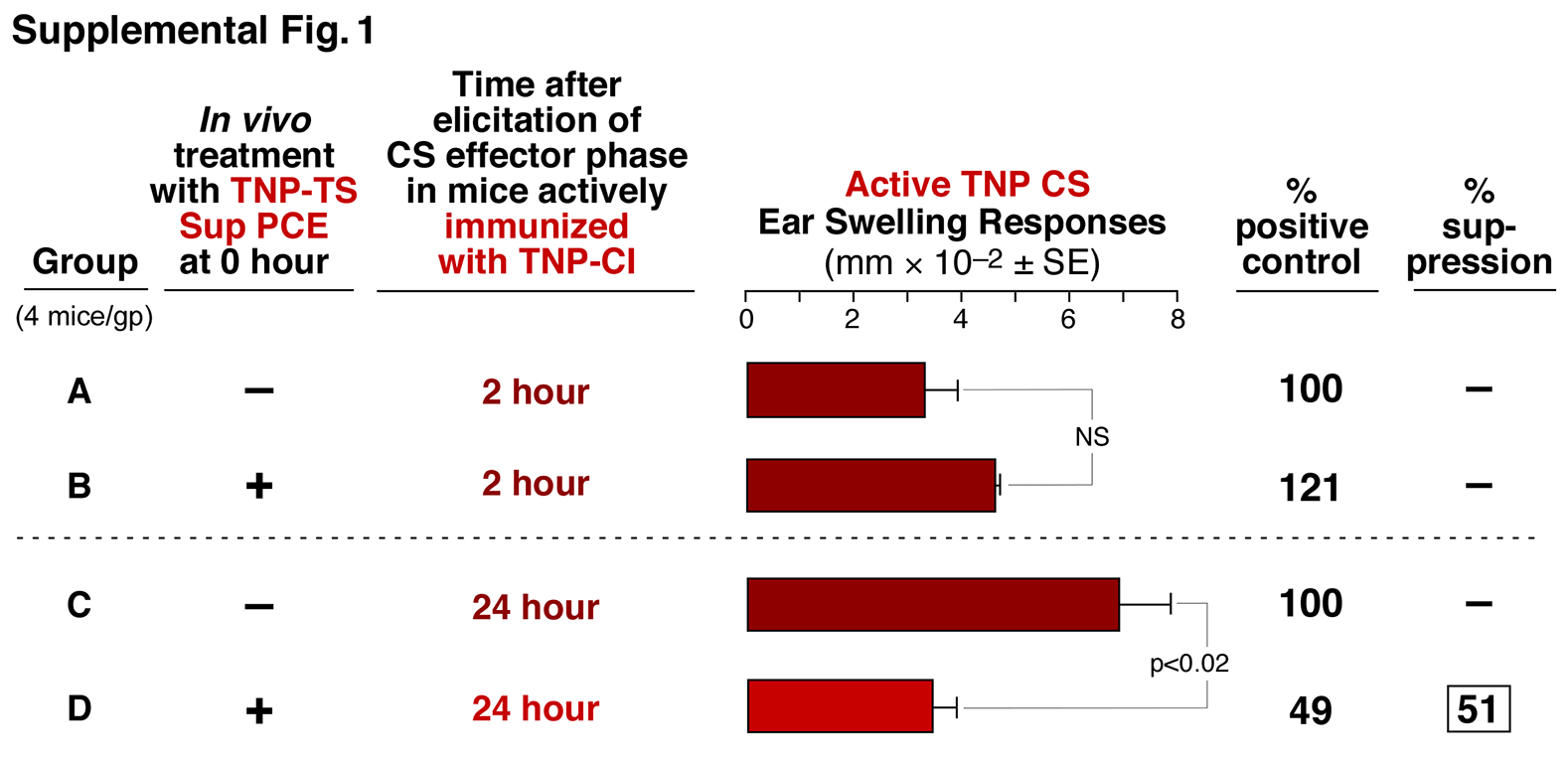

Supplement: S1 Fig — Treatment of actively TNP contact sensitized mice with PCE from TNP Ts Sup results in suppression of the classical 24h component of elicited CS, but not the early 2h CS component (Group D vs B). (TIF) [file pone.0122991.s001.tif]

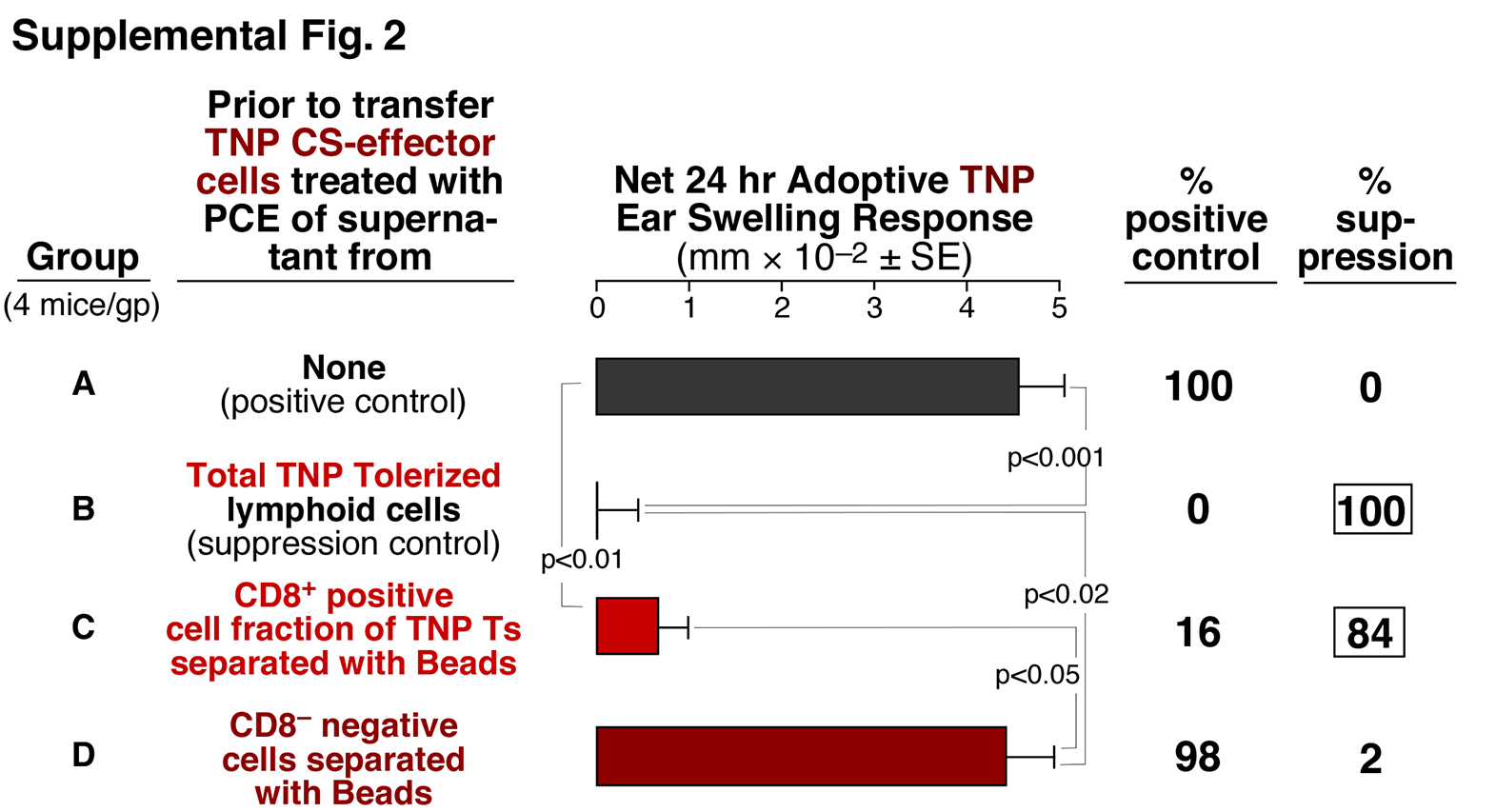

Supplement: S2 Fig — Only PCE from the fraction of Ts Sup isolated from magnetic bead separated CD8+ cells from TNP tolerized mice suppresses TNP-CS-effector cell transfer (Group C vs D). (TIF) [file pone.0122991.s002.tif]

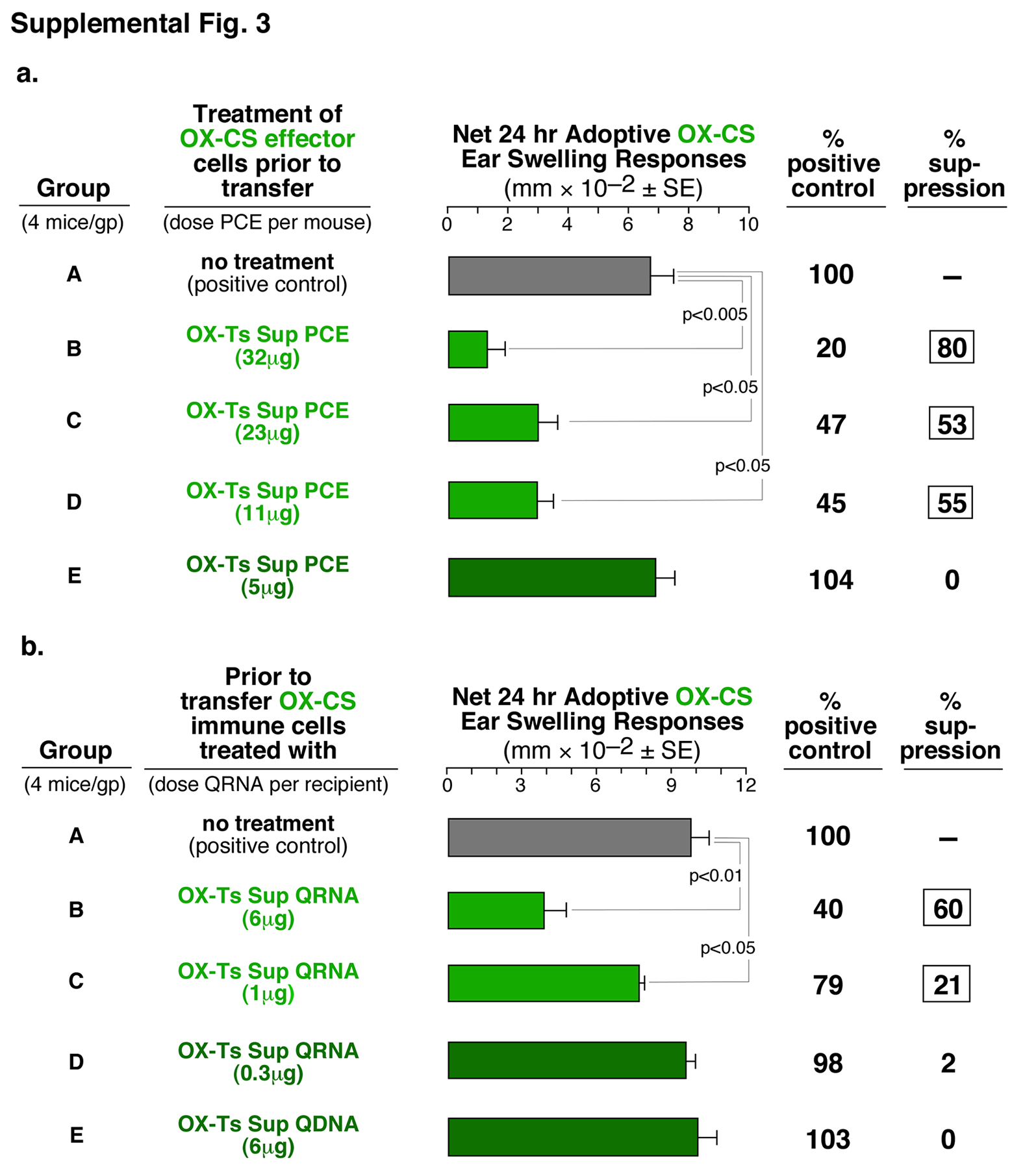

Supplement: S3 Fig — a. Decreasing doses of PCE from OX Ts Sup regressively suppress adoptive CS responses down to a dose of 11μg per recipient (Groups B to D). b. Similarly, dose-response treatment with QRNA from OX Ts Sup showed that QRNA suppresses CS response down to a dose of 1μg per recipient (Group C). (TIF) [file pone.0122991.s003.tif]

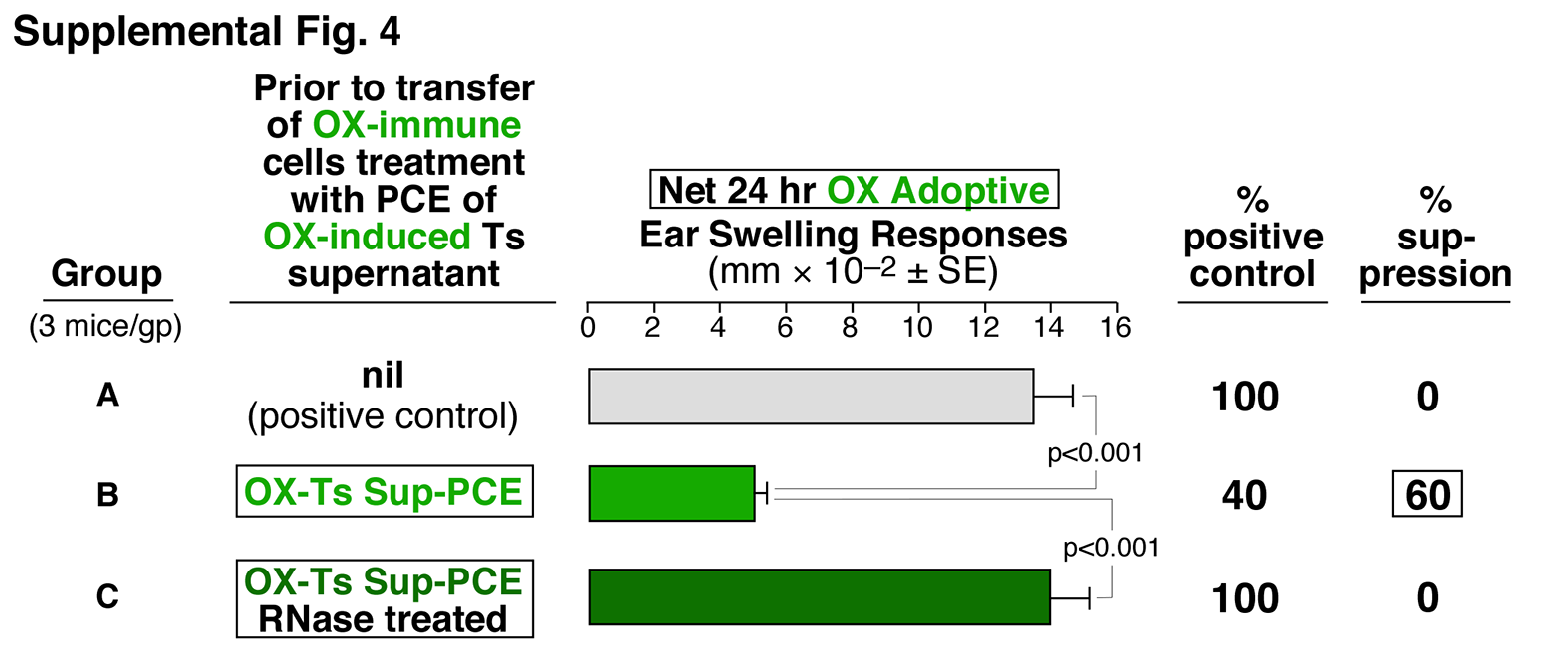

Supplement: S4 Fig — Treatment with RNase of PCE from OX Ts Sup eliminates suppression of adoptively transferred CS response (Group C vs B). (TIF) [file pone.0122991.s004.tif]

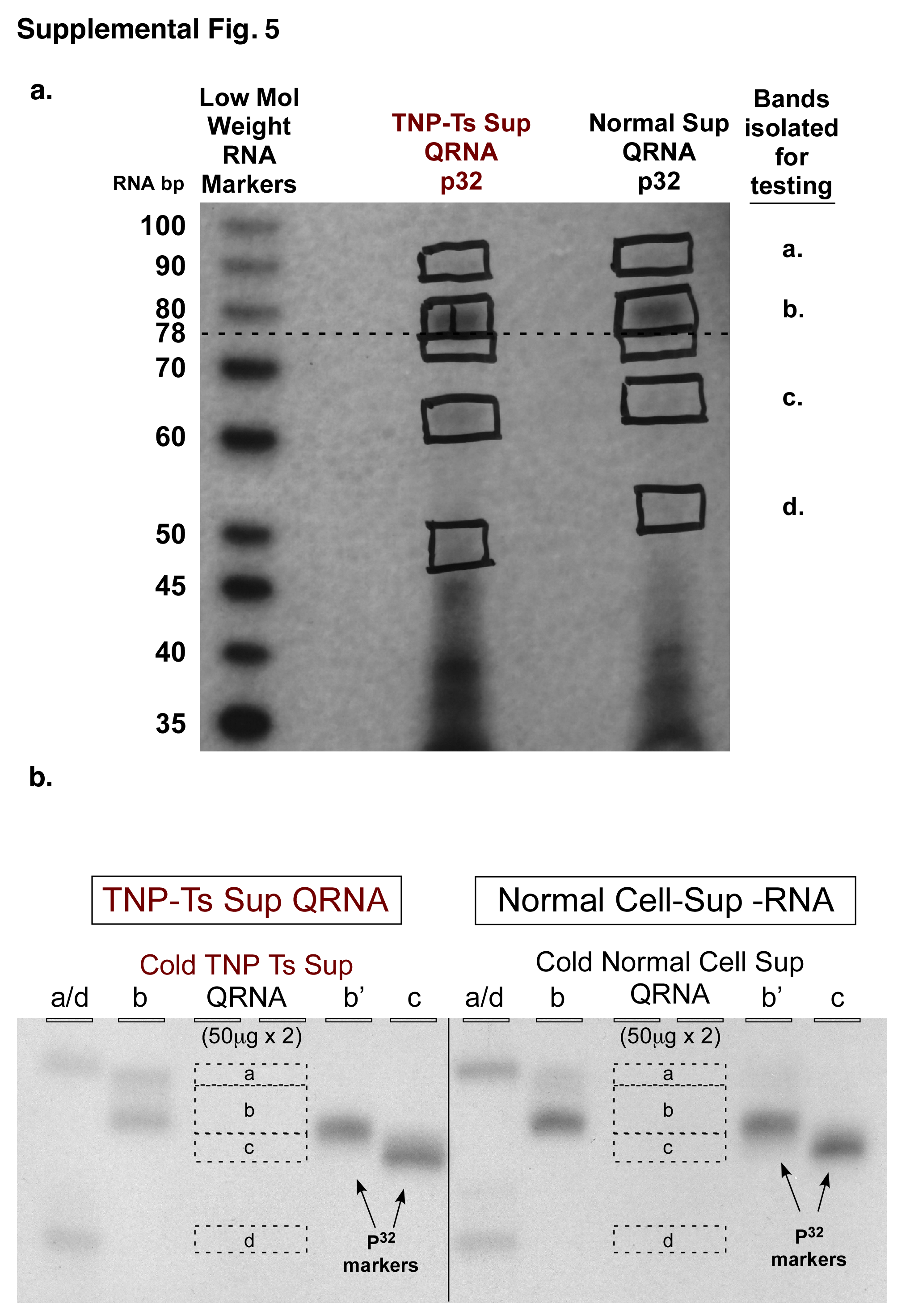

Supplement: S5 Fig — a . Radioautographic visualization of electrophoretically separated fractions of P32 labeled QRNA of TNP Ts Sup on a 12% polyacrylamide sizing gel. b . Preparative fractions of unlabeled QRNA from TNP Ts and Nl Cell Sup to test in adoptive transfer of CS in vivo and inhibition of HT-2 cell responsiveness to IL-2 in vitro. (TIF) [file pone.0122991.s005.tif]

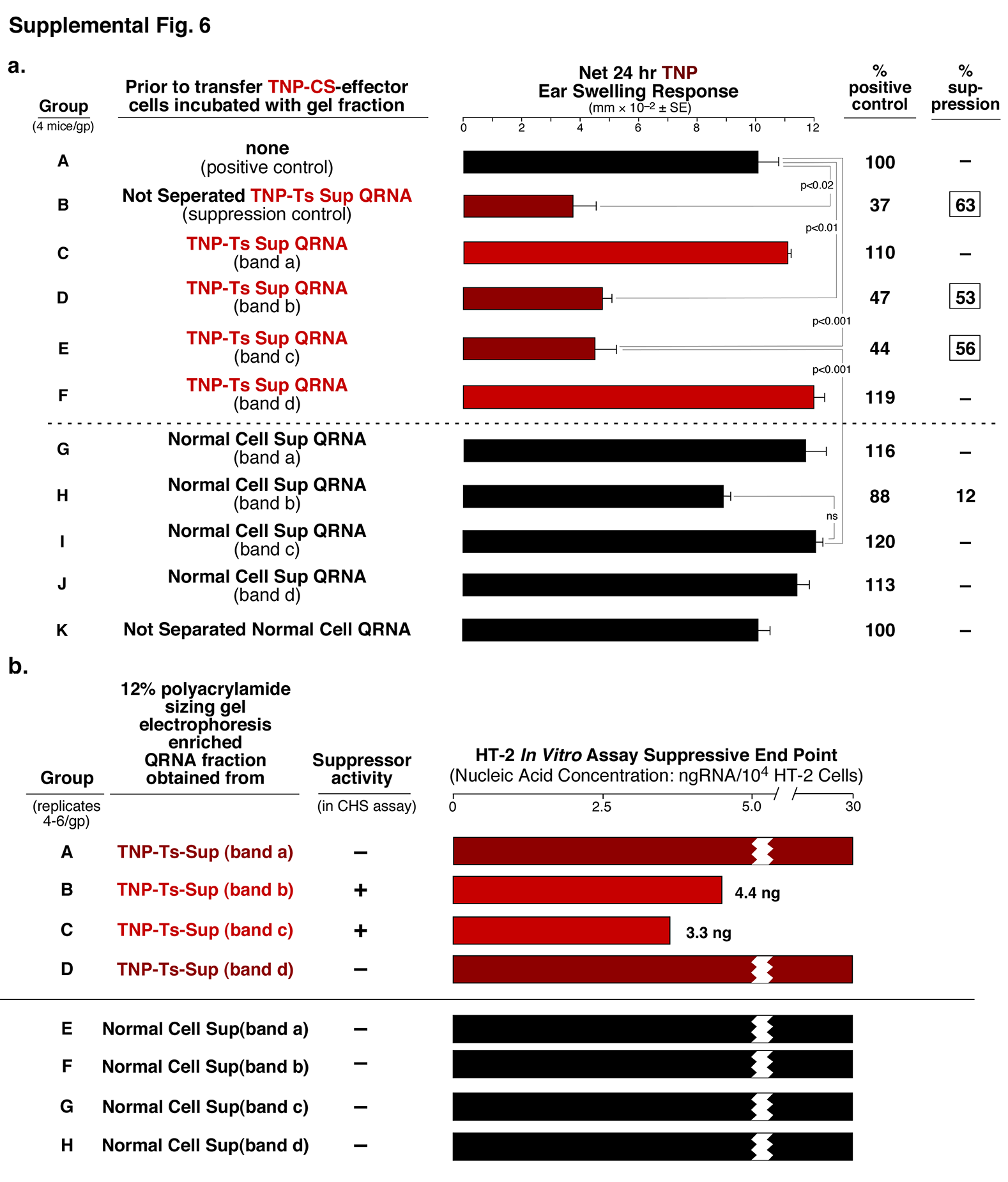

Supplement: S6 Fig — a. Only RNA fractions of about 75bp from TNP Ts Sup QRNA separated on sizing gel inhibit adoptively transferred TNP-CS-effector cells (Groups D and E) compared to unseparated TNP Ts Sup QRNA (Group B), whereas Nl Cell Sup QRNA fractions were not suppressive (Groups H and I). b. Similarly, TNP Ts Sup-derived QRNA (about 75bp) fractions separated on sizing gel inhibit IL-2 dependent viability of HT-2 cells (Groups B and C), while related fractions of Nl Cell Sup were not suppressive (Groups F and G). (TIF) [file pone.0122991.s006.tif]

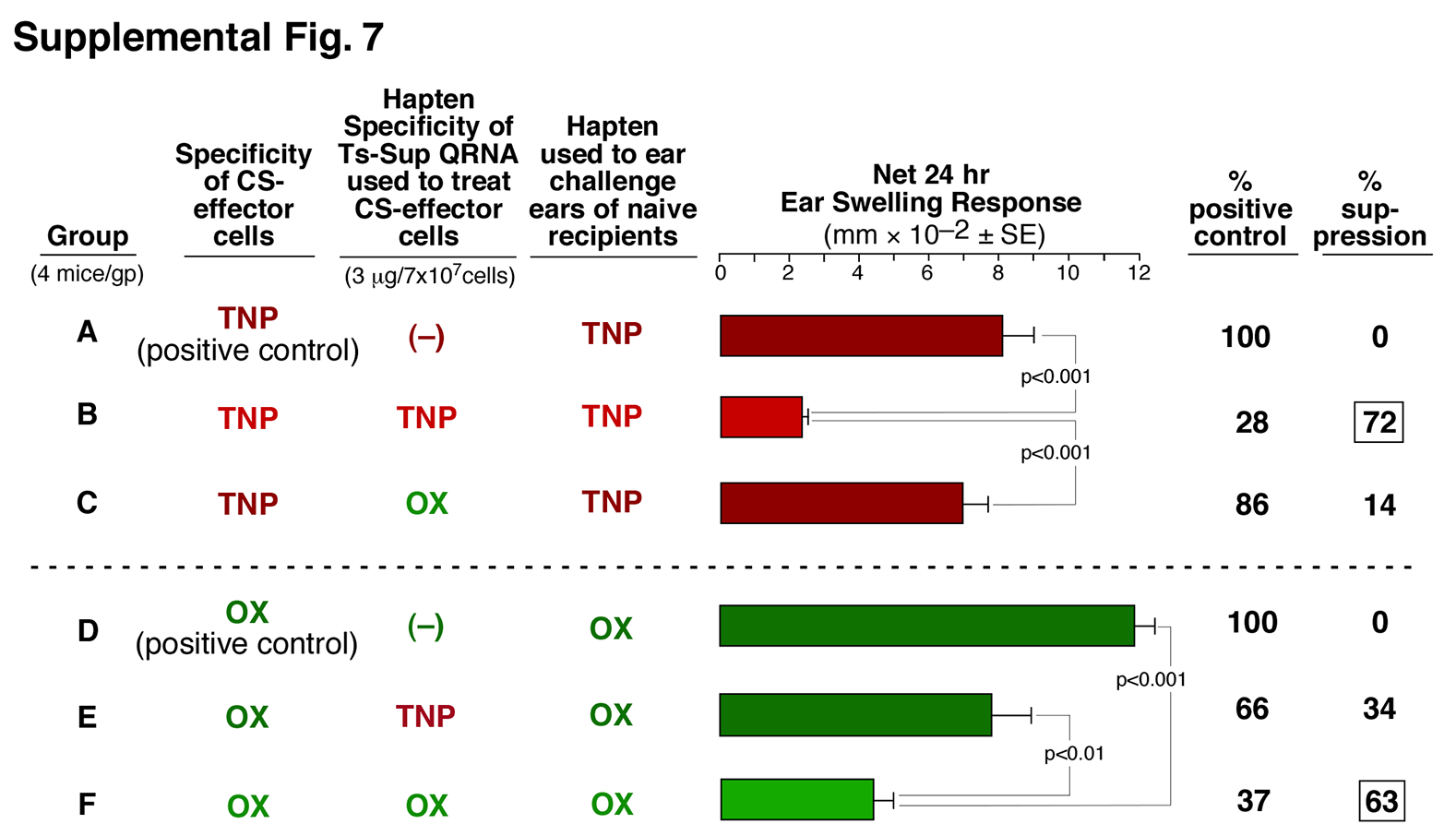

Supplement: S7 Fig — TNP vs OX-specific CS-effector cells were strongly suppressed by QRNA from Ts cell induced by respective homologous hapten (Groups B and F), whereas QRNA from heterologous hapten induced Ts cells was significantly less effective (Groups C and E). (TIF) [file pone.0122991.s007.tif]

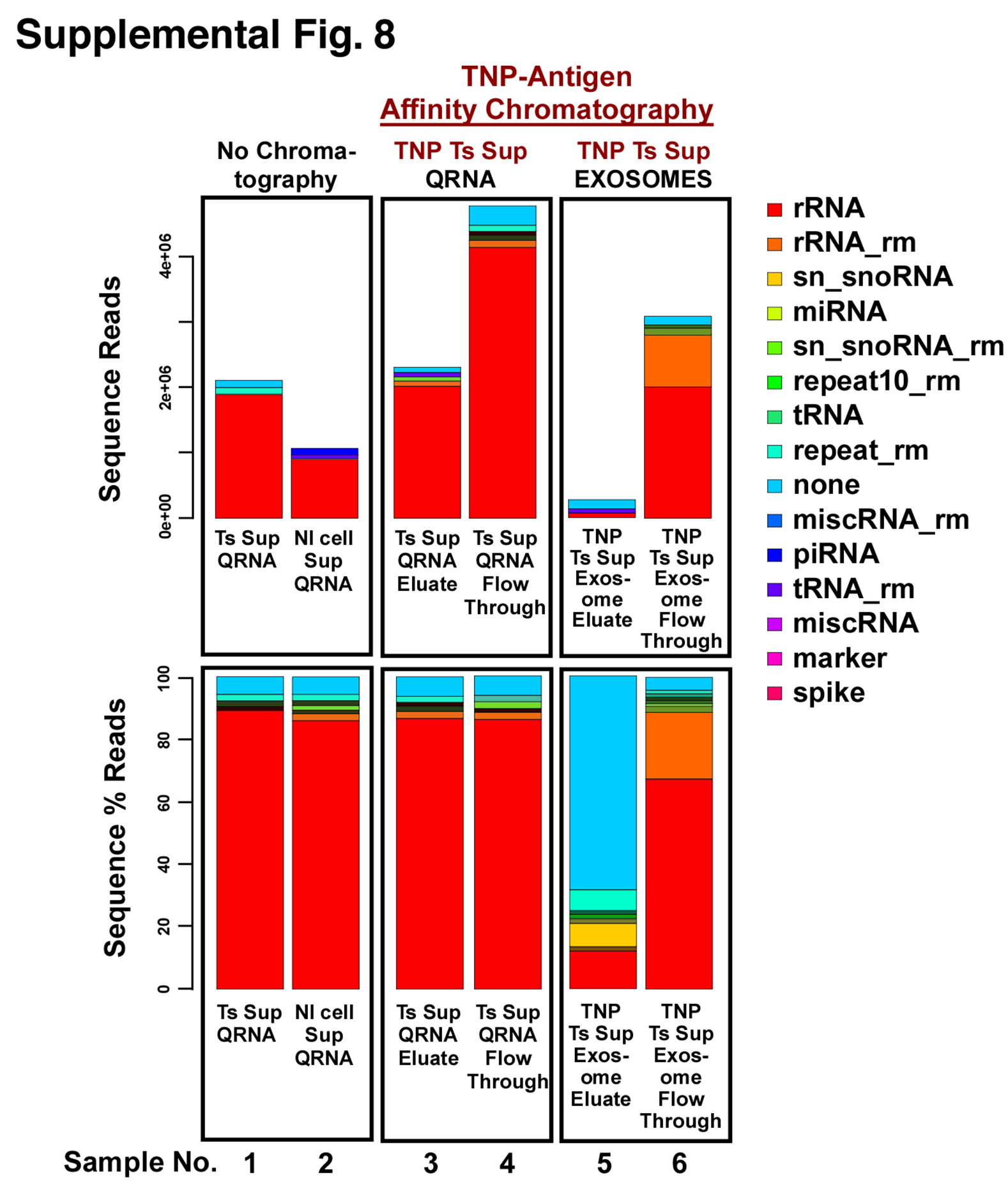

Supplement: S8 Fig — QRNA from TNP Ts Sup before and after separation with TNP-affinity column chromatography express almost identical annotations of RNA subtypes and thus very similar transcriptomes (samples 3 and 4), comparable to QRNA from Nl Cell Sup (Sample 2), whereas TNP-affinity separation of exosomes from TNP Ts Sup fractionates exosomes into Ag-binding (suppressive) and non-binding (non-suppressive) fractions with very different transcriptomes (Sample 5 vs 6). (TIF) [file pone.0122991.s008.tif]

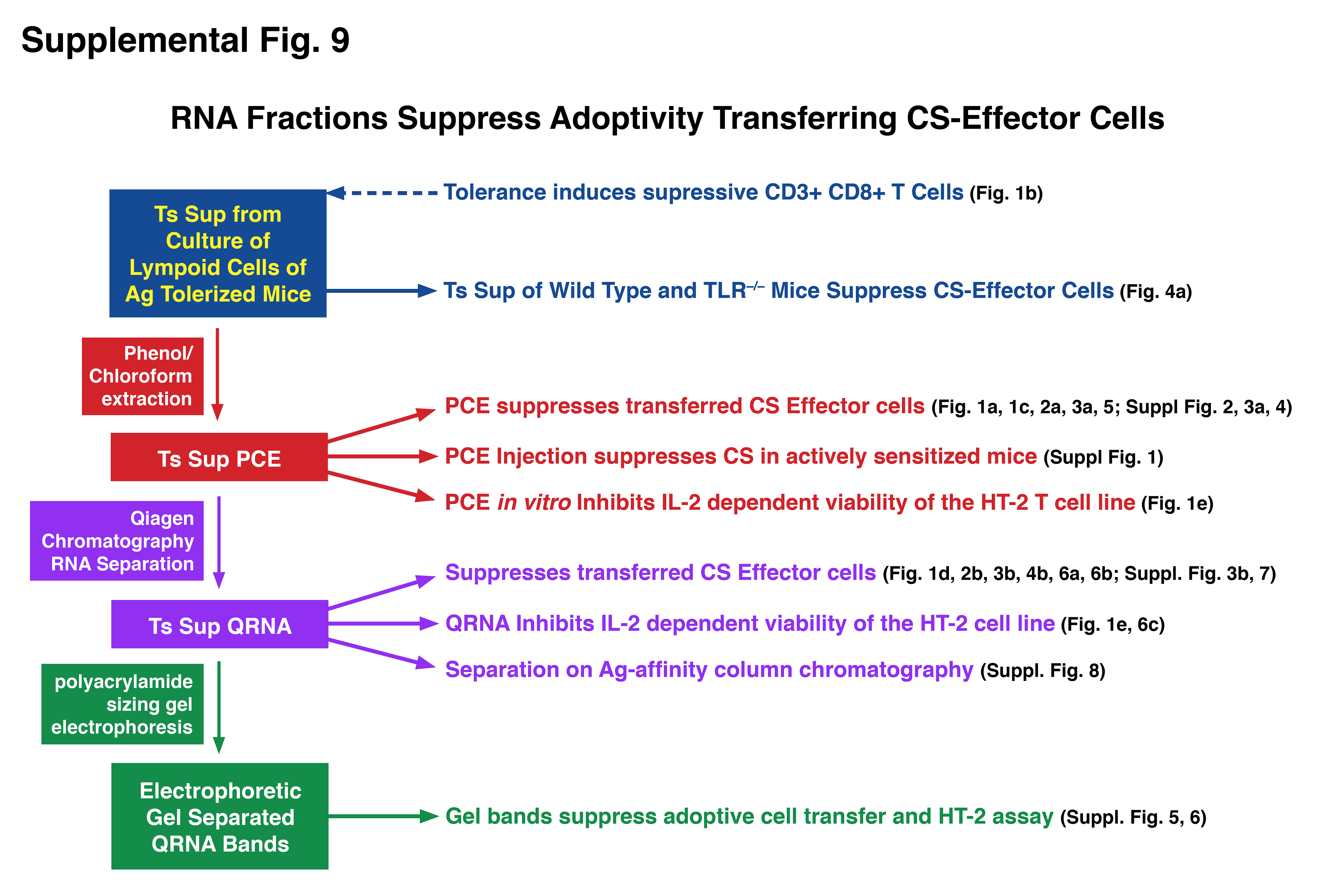

Supplement: S9 Fig — Figure shows the scheme of further preparation of Ts Sup and experimental usage of resulting fractions. (TIF) [file pone.0122991.s009.tif]
